# Comparative Analysis of CDPK Family in Maize, *Arabidopsis*, Rice, and Sorghum Revealed Potential Targets for Drought Tolerance Improvement

**DOI:** 10.3389/fchem.2017.00115

**Published:** 2017-12-19

**Authors:** Shikha Mittal, Mallana Gowdra Mallikarjuna, Atmakuri R. Rao, Prashant A. Jain, Prasanta K. Dash, Nepolean Thirunavukkarasu

**Affiliations:** ^1^Division of Genetics, Indian Agricultural Research Institute (ICAR), New Delhi, India; ^2^Centre for Agricultural Bioinformatics, Indian Agricultural Statistics Research Institute (ICAR), New Delhi, India; ^3^Department of Computational Biology & Bioinformatics, Sam Higginbottom University of Agriculture, Technology, and Sciences, Allahabad, India; ^4^ICAR-National Research Centre on Plant Biotechnology, New Delhi, India

**Keywords:** CDPK, differential expression, drought, 3-D protein structure, functional mechanism

## Abstract

Calcium dependent protein kinases (CDPKs) play significant role in regulation of plant growth and development in response to various stresses including drought. A set of 32 CDPK genes identified in maize were further used for searching of orthologs in the model plant *Arabidopsis* (72) and major food crops such as rice (78) and sorghum (91). We comprehensively studied the phylogenetic relationship, annotations, gene duplications, gene structure, divergence time, 3-D protein structures and tissue-specific drought induced expression of CDPK genes in all four species. Variation in intron frequency in the studied species was one of the reasons for the functional diversity of CDPK genes to various stress responses. Protein kinase and protein kinase C phosphorylation site domains were the most conserved motifs identified in all species. Four groups were identified from the sequence-based phylogenetic analysis, in which maize CDPKs were clustered in group III. Expression data showed that the CDPK genes were highly expressed in leaf of maize, rice, and sorghum whereas in *Arabidopsis* the maximum expression was observed in root. The expression assay showed 5, 6, 11, and 9 were the commonly and differentially expressed drought-related orthologous genes in maize, *Arabidopsis*, rice, and sorghum, respectively. 3-D protein structure were predicted for the nine genes (*Arabidopsis*: 2, maize: 2, rice: 3, and sorghum: 2) showing differential expression in at least three species. The predicted 3-D structures were further evaluated and validated by Ramachandran plot, ANOLEA, ProSA, and Verify-3D. The superimposed 3-D structure of drought-related orthologous proteins retained similar folding pattern owing to their conserved nature. Functional annotation revealed the involvement of CDPK genes in various pathways such as osmotic homeostasis, cell protection, and root growth. The interactions of CDPK genes in various pathways play crucial role in imparting drought tolerance through different ABA and MAPK signaling cascades. These selected candidate genes could be targeted in development of drought tolerant genotypes in maize, rice, and sorghum through appropriate breeding approaches. Our comparative experiments of CDPK genes could also be extended in the drought stress breeding programmes of the related species.

## Introduction

Plants are evolved with numerous signaling pathways to adapt with stress environments. The transfer of stress signals in plants is mediated by various primary and secondary messengers. Calcium (Ca^2+^) acts as a universal secondary messenger in signaling pathways and regulates growth and development (Boudsocq and Sheen, 2013). Several stimuli including hormones, elicitors (Romeis et al., 2001), light (Frattini et al., 1999), and different abiotic stresses change the intracellular Ca^2+^ concentration (Sanders et al., 2002) Calcium binding proteins or calcium sensors (calmodulin, calmodulin like proteins, calcineurin B-like proteins, and calcium dependent protein kinases) recognize the changes in Ca^2+^ concentration and results in downstream expression events (Sanders et al., 1999).

Among these sensors, CDPKs serve as special sensors as they can directly convert upstream Ca^2+^ signals into downstream protein phosphorylation events as they have both sensing and responding behavior owing to the presence of CaM like and protein kinase domains (Poovaiah et al., 2013). CDPK's have been identified all over plant kingdom including some protozoan's (Harper and Harmon, 2005).

CDPKs possess a variable N-terminal domain which often contains myristoylation sites for membrane association (Cheng et al., 2002). The catalytic Ser/Thr kinase domain or protein kinase domain contains ATP-binding site, which plays an important role in the function of CDPK. An auto-inhibitory region which acts as an auto-inhibitor in the absence of Ca^2+^ stimulation and blocks the enzyme active site, and camodulin-like domain which contains EF hands for calcium ion binding (Sanders et al., 2002) and C-terminal domain (Hrabak et al., 2003).

Genome wide analysis led to identification of CDPK proteins in various plant species such as rice, wheat, *Arabidopsis*, poplar, grapes etc. (Chen et al., 2013; Zuo et al., 2013). CDPK proteins play major biological and functional roles *viz*., root development (Ivashuta et al., 2005), pollen tube elongation (Myers et al., 2009), cell differentiation, programmed cell death (Lee et al., 2003), hormone signaling (Liese and Romeis, 2013).

Drought is considered as one of the major abiotic stress responsible for restraining crop production and productivity in the developing world (Aravind et al., 2017). Drought stress decreases the water potential of different parts of plant, closing of stomata through increased accumulation of abscisic acid (ABA) and leads to reduced photosynthesis and decreased biomass and grain yield (Shikha et al., 2017; Thirunavukkarasu et al., 2017; Van Gioi et al., 2017). CDPKs have been shown to be involved in abiotic stress tolerance in various plants. The first CDPK activities were reported ~25 years ago in pea shoot membranes (Hetherington and Trewavas, 1982). CDPKs were often recognized as positive regulators of abiotic stress responses and the over-expression of the respective kinase leads to plants with improved stress tolerance (Asano et al., 2012; Boudsocq and Sheen, 2013). A positive regulatory outcome of CDPKs may be explained by the enhanced expression of ABA-responsive genes in drought stress signaling (Xu et al., 2010).

Previous studies also reported the involvement of CDPK genes with drought tolerance enhancement. Over-expression of *OsCPK7* and *AtCPK6* in rice and Arabidopsis, respectively led to enhanced drought tolerance (Saijo et al., 2000; Xu et al., 2010). Similarly, *AtCPK10* under drought stress regulates the stomatal movement through interaction with *HSP1* in *Arabidopsis* (Zou et al., 2010). Evidences also indicated the participation of *OsCPK9* and *AtCPK10* in ABA-responsive drought tolerance (Zou et al., 2010; Wei et al., 2014). *ZoCDPK1* from ginger was found to promote the drought tolerance by improving growth and photosynthesis in *Nicotina tabacum* (Vivek et al., 2013). *OsCPK10* played a role in drought stress tolerance by defending cellular membranes through an enhanced capacity to detoxify ROS (Kumar et al., 2014; Nakabayashi et al., 2014; Fang et al., 2015; Yin et al., 2015).

Various studies have reported that the CDPK genes not only behave as positive regulator of abiotic stress signaling but also a negative regulator. For example, *AtCPK23* improved the drought tolerance in Arabidopsis by behaving as a negative regulator of abiotic stress signaling (Ma and Wu, 2007). Similarly, *ZmCPKs* and *OsCPKs* negatively regulate abiotic stress (Wang et al., 2013). In Arabidopsis, *AtCPK12* has been categorized as a negative ABA signaling pathway regulator (Zhao et al., 2011). In another report it has been observed that *CIPK15* interacts with CBL1 as a negative regulator of ABA signaling pathway (Guo et al., 2001). The down-regulation of *ZmCPK14* after the ABA treatment in maize suggesting that *ZmCPK14* might act as a negative regulator in ABA signaling (Kong et al., 2013).

Our main focus of the study was to characterize the CDPK genes in maize and to find its orthologs in model plant Arabidopsis and major food crops such as rice and sorghum and study their role in drought tolerance. Our objectives were to predict the putative orthologs of maize CDPK genes in *Arabidopsis*, rice, and sorghum species, to comprehensively characterize the genes using different *in-silico* tools, to determine the expression pattern of CDPK orthologous genes using public data, to superimpose the predicted 3-D protein structure of selected drought-related genes to determine the functional conservation and differentiation, and to explain the functional role of CDPK genes in drought tolerance. To our knowledge, this is the first study in which 3-D structures of drought-related CDPK orthologs were compared and superimposed to understand the structural variation of CDPK gene family in different species. Our investigation also proposed a working hypothesis about the involvement of CDPK genes in various pathways in controlling drought tolerance.

## Materials and methods

### Identification of CDPK gene families

The CDPK genes for maize were retrieved from ProFITS (Protein Families Involved in the Transduction of Signalling) database (http://bioinfo.cau.edu.cn/ProFITS/) and searched for domains using Pfam (http://pfam.xfam.org/). Using the compiled domain ID obtained from Pfam, the Phytozome tool Biomart (http://www.phytozome.net) was used for obtaining 3,332 hits of the CDPK genes in *Arabidopsis*, rice and sorghum. The protein sequences were retrieved from MaizeGDB (http://www.maizegdb.org/) for maize, TAIR (https://www.arabidopsis.org) for *Arabidopsis*, Rice Genome Annotation Project (http://rice.plantbiology.msu.edu) for rice and from Ensembl plants (http://plants.ensembl.org/index.html) for sorghum. The retrieved protein sequences were then subjected to protein BLAST with reference database of *Arabidopsis* and the query file composed of maize, rice, and sorghum protein sequences. Out of 3,332 hits, 273 CDPK proteins were selected on the basis of their domains for further analysis on the basis of percent identity ≥75% and significant *E*-value (1e-6) from the BLAST output (Wu et al., 2017).

### Physiochemical properties of CDPK proteins

The primary information on CDPKs *viz*., chromosome and gene location was retrieved from Ensembl plants (http://plants.ensembl.org/index.html). To get more information about the nature of proteins, we predicted isoelectric point (*pI*), molecular weight, instability index, and grand average of hydropathy (GRAVY) by using ProtParam tool available on Expert Protein Analysis System (ExPasy) proteomics server (http://web.expasy.org/compute_pi) (Gasteiger et al., 2003).

### Gene structure analysis

To identify the gene structure or exon-intron structure of the 273 CDPKs genes, the genomic sequences were aligned with their corresponding coding sequences in GSDS 2.0 server (http://gsds.cbi.pku.edu.cn). The genomic and coding sequences of the genes were extracted from Phytozome BioMart (http://www.phytozome.net).

### Gene ontology (GO) annotation

To provide GO annotation, protein sequences of 273 CDPK proteins were retrieved using Phytozome Biomart (http://www.phytozome.net) and were searched using BLAST. These BLAST results were then used as input to Blast2GO (https://www.blast2go.com/) to assign Gene Ontology (GO) terms i.e., biological process (BP), molecular function (MF), and cellular component (CC).

### Phylogenetic analysis

Multiple sequence alignment of the full-length protein sequences of all four species was used to generate the phylogenetic tree of CDPK proteins through ClustalW (Thompson et al., 1994). Phylogenetic tree was created by using Neighbour-Joining method with 1,000 rapid bootstrap replicates with the help of MEGA v6.06 (Tamura et al., 2013). The developed phylogenetic tree was visualized using iTOL (http://itol.embl.de).

### Gene duplication, orthologs, and paralogs identification

Orthologous genes were identified using BLAST search against *Arabidopsis*, maize, rice, and sorghum. Potential orthologs were identified by performing reciprocal blast of proteins whose sequence identity was >75%. Tandem and segmental duplications was also identified by performing BLAST. Paralogous pairs located on the same chromosome either adjacent or separated by five or fewer genes were considered to be duplicated by tandem duplication. Paralogous pairs within known genomic duplication blocks were assigned as duplicates through segmental duplication (Guo et al., 2013). Synonymous and non-synonymous substitution rates for orthologous and paralogous genes were carried out using PAL2NAL server (http://www.bork.embl.de/pal2nal/).

### Amino acid motif prediction

Conserved motifs among CDPK proteins of *Arabidopsis*, maize, rice, and sorghum were predicted using Multiple maximization for Motif Elicitation analysis tool (MEME v4.10.2; http://meme-suite.org/; Bailey et al., 2006). The MEME suite was used to search 5 best motifs with minimum width of 12, maximum width of 60 and *E* < 0.01. Myristoylation and Palmitoylation sites were predicted using PlantsP (http://plantsp.genomics.purdue.edu/myrist.html) and CSS-Palm 3.0 (Ren et al., 2008) respectively while N-terminal acylation prediction was done using NetAcet 1.0 (Kiemer et al., 2005). EF-hand domains for all the sequences were predicted using SMART (http://smart.embl-heidelberg.de/).

### Gene expression analysis

The drought-related expression of CDPK genes was examined using available published water deficit microarray studies. For *Arabidopsis*, GEO datasets GSE76827, GSE55431, and *Arabidopsis* EFP browser containing data of root, shoot, and leaves in different time point and days were used. For maize, drought expression data of root, stem leaf, cob, and tassel was collected from different studies having GEO datasets accession number GSE40070, GSE71377, and GSE71723 while in case of rice, expression data for leaf and root tissue was collected from GSE38130, GSE65022, and Rice EFP browser. For sorghum, expression data for root and leaf was collected from GSE30249 and GSE80699.

### Secondary and tertiary structure prediction of CDPK proteins

The secondary as well as the 3-D structure of the selected nine drought-responsive orthologous CDPK proteins of *Arabidopsis*, maize, rice, and sorghum were predicted using an online protein structure prediction tool Phyre2 (http://www.sbg.bio.ic.ac.uk/phyre2/html/page.cgi?id=index), which is based on distant homology recognition techniques with secondary structure prediction and domain analysis of each CDPK protein model (Kelley and Sternberg, 2009). The predicted structures were evaluated using Atomic Non-Local Environment assessment (ANOLEA; Melo and Feytmans, 1998), Verify-3D (Eisenberg et al., 1997) and Protein structure analysis (ProSA) program (Wiederstein and Sippl, 2007) and validated using Ramachandran plot. For structural variation analysis of drought-related orthologous CDPK proteins, predicted models of CDPK protein were superimposed in pair-wise manner using the TM-align (http://zhanglab.ccmb.med.umich.edu/TM-align). The predicted structures were visualized in the form of ribbons by PyMol (https://www.pymol.org).

## Result and discussion

### Identification and structural analysis of CDPK family members

Genome-wide analysis led to the identification of 3,332 CDPK genes using EF-hand (Pfam ID: PF00036) and protein kinase (Pfam ID: PF00069) domains belonging to *Arabidopsis*, maize, rice and sorghum. A total of 273 members (Supplementary Table S1) of CDPK family (*Arabidopsis*: 72; maize: 32; rice: 78; and sorghum: 91) were selected for further analysis.

To understand the physico-chemical properties of CDPK proteins, molecular weight (MW), isoelectric point (*pI*), grand hydropathy score (GRAVY) and instability index were predicted for 273 CDPK members. Among 273 members, highest and lowest molecular weight, *pI* and GRAVY were found in rice. Molecular weight varies from 11.93 to 191.41 kDa. The variations in molecular weight was owing to variable number of domains contributing to protein size difference (Hrabak et al., 2003). *pI* is the pH at which a particular molecule carries no net-charge and is important to find out the pH of dependent characteristics proteins (Garcia-Moreno, 2009; Talley and Alexov, 2010). *pI* of CDPKs ranged from 4.11 to 9.99 (Table 1). All 273 proteins showed negative GRAVY scores, suggesting soluble or hydrophilic nature of CDPK proteins (Kyte and Doolittle, 1982). In terms of instability index of these proteins, 43.22% proteins were found to be stable in nature while 56.78% proteins were unstable in nature (Rogers et al., 1986). GRMZM2G154489_P01 protein emerged as the most stable (17.15) while Sb09g021730.1. as the most unstable protein (63.23).

**Table 1:** Physiochemical properties of 273 CDPK genes in *Arabidopsis*, maize, rice and sorghum species.

**Species**	**No. of genes**	**MW (kDa)**			**pI**			**GRAVY**		
		**Min**	**Max**	**Avg**	**Min**	**Max**	**Avg**	**Min**	**Max**	**Avg**
*Arabidopsis*	72	16.85	78.64	50.95	4.11	9.93	7.45	−0.86	−0.11	−0.42
Maize	32	15.97	67.49	54.33	4.56	9.05	6.52	−0.6	−0.14	−0.38
Rice	78	11.95	191.41	51.69	4.11	9.99	7.22	−0.87	−0.04	−0.39
Sorghum	91	12.9	83.01	50.97	4.11	9.71	7.27	−0.8	−0.14	−0.39

### Gene structure analysis

**Figure 1:**
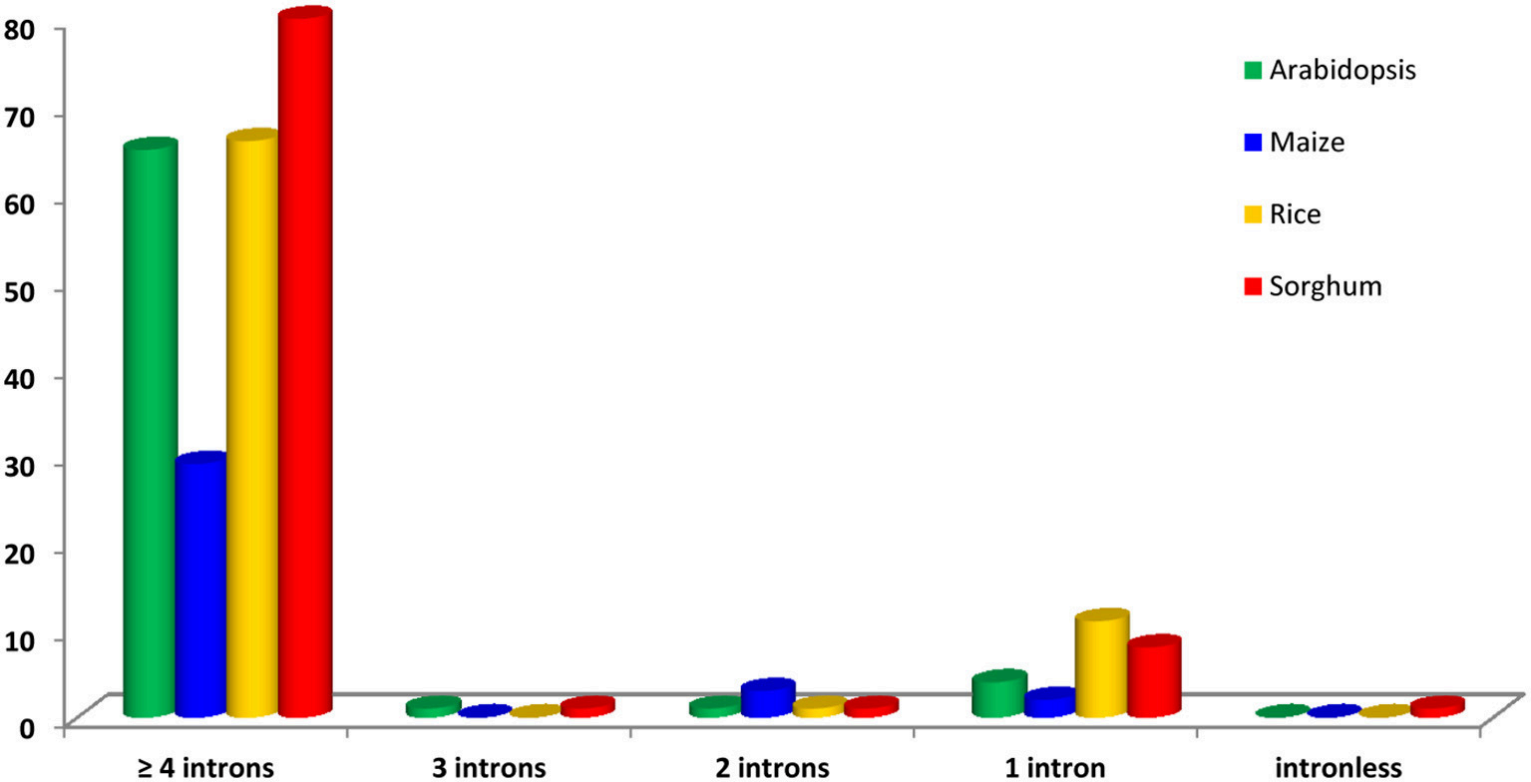
Gene structure distribution of 273 CDPK genes collected from *Arabidopsis*, maize, rice, and sorghum.

Gene structure was examined to get the insights of structural evolution of CDPK genes in different species i.e., *Arabidopsis*, maize, rice, and sorghum. The gene structure analysis showed the details of expansion of gene family and divergence. The number of introns varied from 0 to 18 (Figure 1). We divided the gene structure of 273 proteins into 2 types: Type-I (intron-poor) and Type-II (intron-rich). Those genes in which number of intron were ≤ 5 were considered as intron-poor, while in which number of introns exceeded >5were included in intron-rich type. In type I, only (23.08%) genes were found while many of the genes were included in Type II (76.92%). Several CDPK proteins were intron-rich in their gene structure. In type I, we examined the participation of different species. Out of 4 selected species, maize was the least contributing species (18.75%) in Type I, while sorghum contributed the most (24.18%). In type II, highest number of genes were in maize (81.25%), followed by *Arabidopsis* (77.78%), rice (76.92%), and sorghum (75.82%). Among all the genes, only one gene was found as intron-less (*Sb1599s002010*). Maximum number of introns was also observed in sorghum (*Sb04g036670*) with 18 introns. All the eukaryotes evolved from common ancestor, the extensive loss and gain of introns could have been occurred during the genome evolution which was mediated by selection pressure and population size (Lynch, 2002; Roy and Gilbert, 2006). Furthermore, CDPKs are known to respond various abiotic stress signals through multiple pathways. Therefore, presence of more introns might add the functional diversity to CDPK genes through alternative splicing and exon shuffling (Keren et al., 2010).

### GO annotation

**Figure 2:**
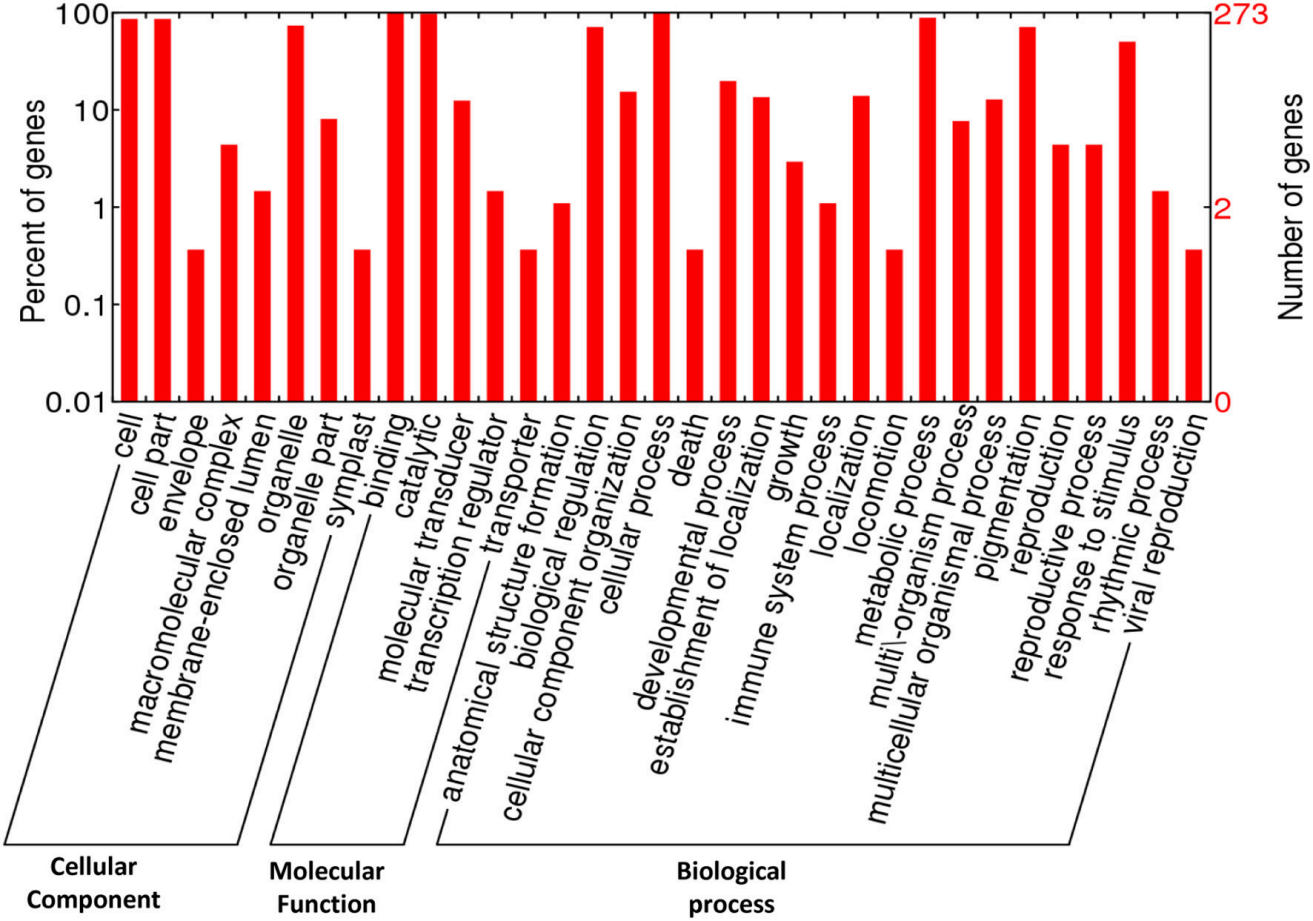
Classification of GO annotation of 273 CDPK genes under different functional categories belonging to *Arabidopsis*, maize, rice, and sorghum.

The GO terms comprise of three categories: (1) Cellular component, (2) Molecular function, and (3) Biological process. The annotated GO terms were categorized into 33 functional groups, including eight GO terms under cellular component, five terms under molecular function, and 20 terms under biological process. In cellular component category-cell, cell part and organelle were top three GO terms, while in case of molecular function, the maximum CDPK genes were found to be involved in binding, catalytic, and molecular transducer activity. In biological processes; cellular process, metabolic process, biological regulation, pigmentation, response to stimulus, and signaling activities were highly enriched (Figure 2).

Out of 273 genes, we identified 46 drought-related genes based on their GO annotation (*Arabidopsis*: 11; maize: 12; rice: 17; and sorghum: 6). Most of these drought-related CDPK genes were related to calcium-dependent kinase, CBL-interacting kinase, cyclin dependent kinase (CDK), Mitogen-activated protein kinase (MAPK), serine threonine kinase activities. CDPKs are highly conserved proteins involved in various functions such as plant growth, development, stress signaling, defense response, and proteasome regulation (Tena et al., 2011; Hubbard et al., 2012). Under drought stress, CDPK acts as a key intracellular protein kinase which converts calcium signals into phosphorylation events. Calmodulin like proteins, one of the most significant calcium sensors, regulates activity of abundant proteins and plays important role in mediating plant stress tolerance. Calmodulin does not have catalytic activity but upon binding with calcium it triggers target proteins which lead to various cellular responses (Bouché et al., 2005). Previous studies reported that MAPK plays significant role in various biotic and abiotic stresses including drought, salt, temperature, wounding, pathogen infection, and hormones (Ichimura et al., 2000; Tena et al., 2001). Under drought stress, role of MAPK signaling had been extremely studied and is known to act downstream of secondary signaling mechanisms (Sun et al., 2015). MAPK, signaling cascades are phosphorylated by specific serine/threonine residues thereby modulating cellular functions. The MAPK cascade having role in stress regulation consists of 3 inter-connected protein kinases-MAP kinase kinase kinase (MAPKKK), MAPKK (MAP kinase kinase), and MAPK.

Similarly, the roles of cyclin-dependent kinases (CDK) and cyclin-dependent kinase inhibitors under drought stress have been reported. CDK are involved in regulation of cell cycle. In water deficit environment, the action of CDK reduces, which increases the period of cell division, thereby decreasing cell divisions per unit time hence leading to reduction in growth of plant and leaves (Granier et al., 2000). In our study, among refined drought-related genes, only LOC_Os07g02350.1 has been annotated as casein kinase II (CK2). Recently CK2 has been identified as negative regulator of SnRK2 (Vilela et al., 2015). CK2, a serine/threonine kinase found in all eukaryotes is distinctive in nature as it can use either ATP or GTP as phosphoryl donors (Vilela et al., 2015). CK2 is found to be involved in response to various biotic and abiotic stresses, plant growth and development, regulation of cell cycle, hormone response, time of flowering, light-regulated gene expression, and DNA repair (Mulekar and Huq, 2014; Vilela et al., 2015). CK2 acts as an ABA regulator because *ck2*α mutants are hypersensitive to ABA concerning seed germination, cotyledon greening, and stomatal opening (Mulekar and Huq, 2014; Wang et al., 2014).

**Figure 3:**
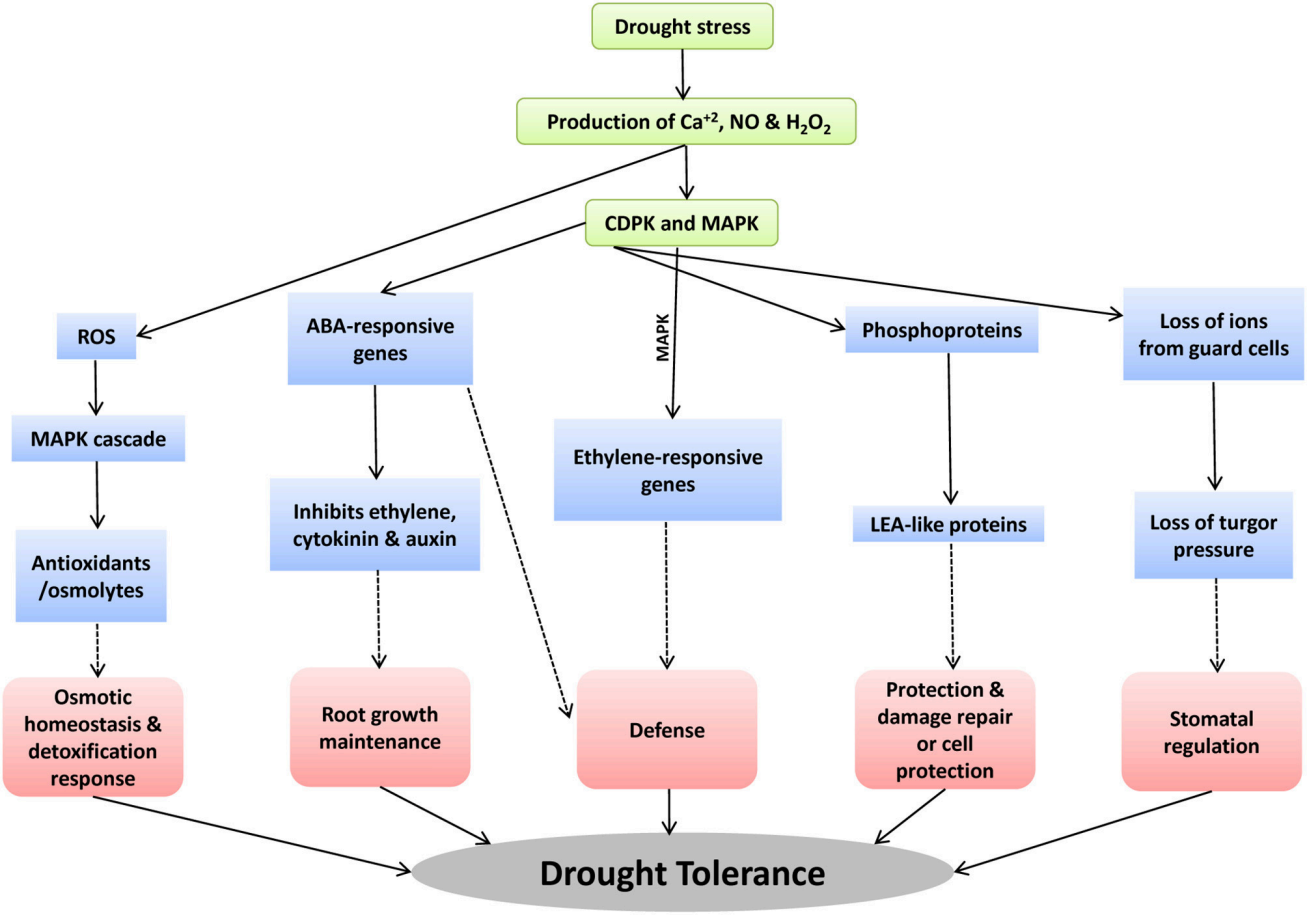
The response of CDPK genes during drought stress and the possible drought tolerant mechanisms controlled by the CDPK genes.

CDPKs play significant role in different drought tolerance mechanisms such as stomatal regulation, root growth maintenance, defense, and cell damage and repair (Figure 3). For example, abiotic stress accompanies the ROS formation such as hydrogen peroxide, hypochlorite ion etc. MAPK pathways triggers this ROS signaling pathway by the receptors or sensors which further activate the osmolyte/antioxidants production which are very important for osmotic homeostasis under drought stress tolerance (Wang et al., 2003). Secondly, increase in concentration of ABA triggers H_2_O_2_, NO and Ca^+2^ signaling (Courtois et al., 2008; Neill et al., 2008). Further, specific CDPK and MAPK regulate the expression of ABA-responsive genes by the activation of transcription factors (García-Mata and Lamattina, 2001). These ABA-responsive genes inhibit the levels of ethylene, cytokinin, and auxin which favor the maintenance of root growth for drought stress tolerance. Thirdly, many of the previous studies have supported the regulation of stomatal aperture by NO and turgid cells under drought stress (Tanaka et al., 2005; Bright et al., 2006; Munemasa et al., 2007; Closure et al., 2011). The CDPK pathways induce the expression of LEA-proteins such as dehydration responsive element which further helps in stress damage control and repair.

### Multiple sequence alignment and phylogenetic analysis

**Figure 4:**
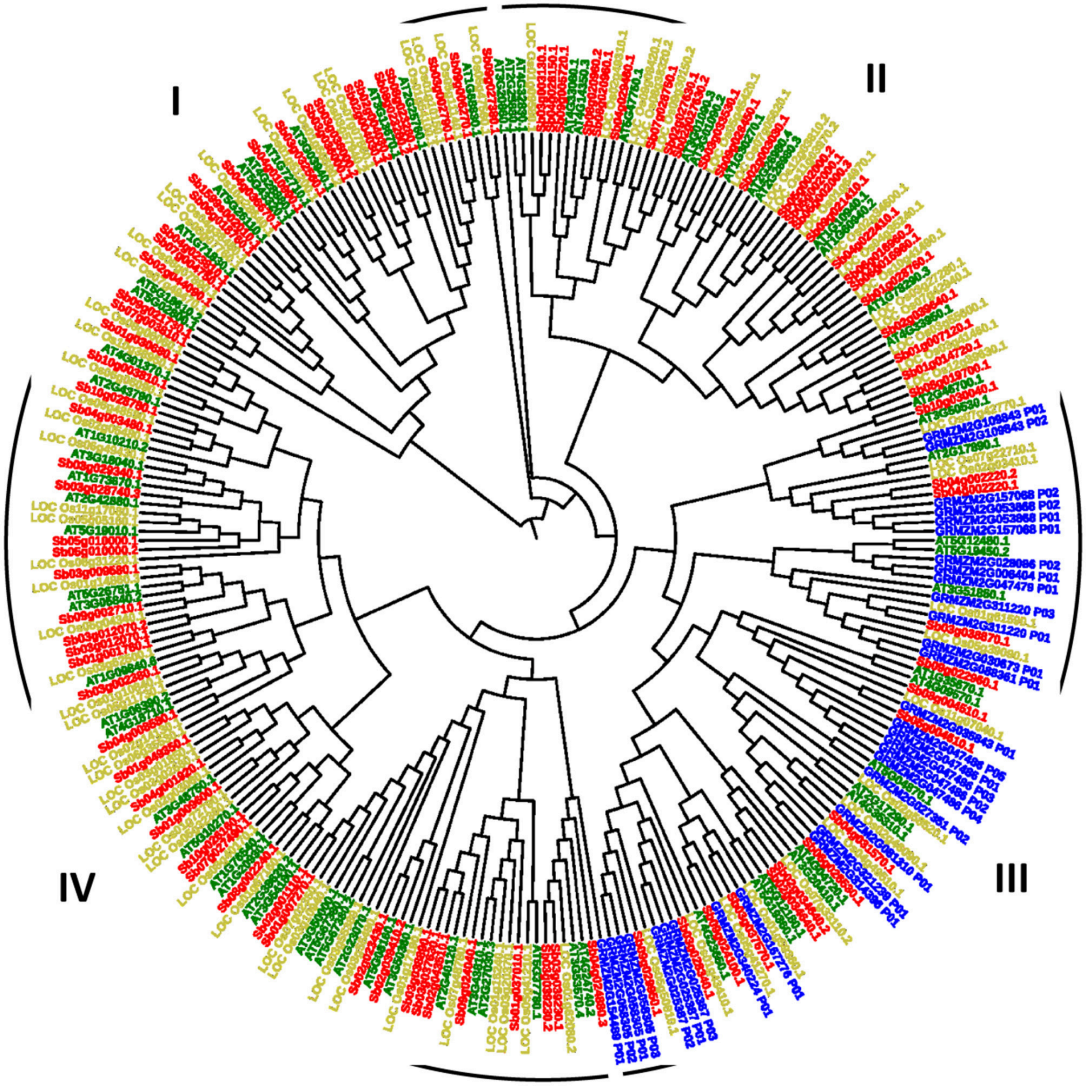
A rooted pylogenetic tree of 273 CDPK genes generated using MEGA software with 1,000 bootstrapping replicates and divided into four groups- I, II, III, and IV where green, blue, golden, and red color represents *Arabidopsis*, maize, rice, and sorghum species.

To know the evolutionary relationship among the model plant (*Arabidopsis*) and major food crops (rice, maize, and sorghum), a rooted phylogenetic tree was generated (Figure 4). Phylogenetic analysis revealed four groups in which Group IV was the largest group consisting of 33 members of rice, 30 members of *Arabidopsis* and 35 members of sorghum species while Group I was the smallest one with 41 members (*Arabidopsis*: 11; rice: 12; sorghum: 18). Similar kind of grouping patterns had also been reported in dicot and monocots (Hamel et al., 2014). Further, we divided each group into subgroups according to the conserved domains present in each main group. Group I was divided into-Ia and Ib, II into IIa, IIb, IIc and IId, III into IIIa and IIIb while group IV into-IVa, IVb, IVc. We noticed that CDPK genes of maize were clustered distinctly in group III only except one gene GRMZM2G154489_P01 which was grouped with IVa.

Genes involved in Ib, IIa, IId, IIIa, IVa, IVc subgroups were conserved in having only one copy of protein kinase domain (Supplementary Figure 1A). This domain is a catalytic domain, possesses serine/threonine residues and activation loop and has vital role in CDPK functioning. To avoid loop from unnecessary phosphorylation, activation loop has acidic residues (Harmon et al., 2000; Harper et al., 2004). In subgroup IIIb, protein kinase along with 2 repeats of EF-hand_7 domain was conserved except in three proteins of maize (GRMZM2G028086_P02 and GRMZM2G158305_P02) and one protein of sorghum (Sb02g034640.2). Calcium sensor proteins hold pair of EF-hands, each of which are considered as the crucial functional unit for protein stabilization and facilitate high affinity binding to calcium ions (Luan et al., 2002). When calcium ion binds to EF-hands, it results in conformational changes in the globular structure of CaM proteins, which allows interaction of CaMs with their target proteins (Yamniuk and Vogel, 2005). Similarly, in case of IVb, EF-hand_7 (2 repeats) domain was conserved except in 1 protein of *Arabidopsis* (AT2G40120.1) and 2 proteins of sorghum (Sb02g027630.1, Sb03g038890.1). Subgroup Ia had multiple types of domains. Subgroup IIb had domain-specific protein kinase (2 repeat) while subgroup IIc, UBA, KA1, NAF, and pkinase domains were found (Supplementary Figure 1B). The CDPK evolutionary analyses revealed that the division of phylogenetic groups corresponds well with their pfam domain structures and sequence conservation.

The reason behind the maize genes available in a different conserved group is that these genes might have similar functional relationship. While the other genes of different species present in this group represents the orthologous relationship. By examining their domains, it was noticed that all these genes consist of same type of domains so they belong to the same group.

### Gene duplication, orthologs, paralogs, and Ks dating

**Figure 5:**
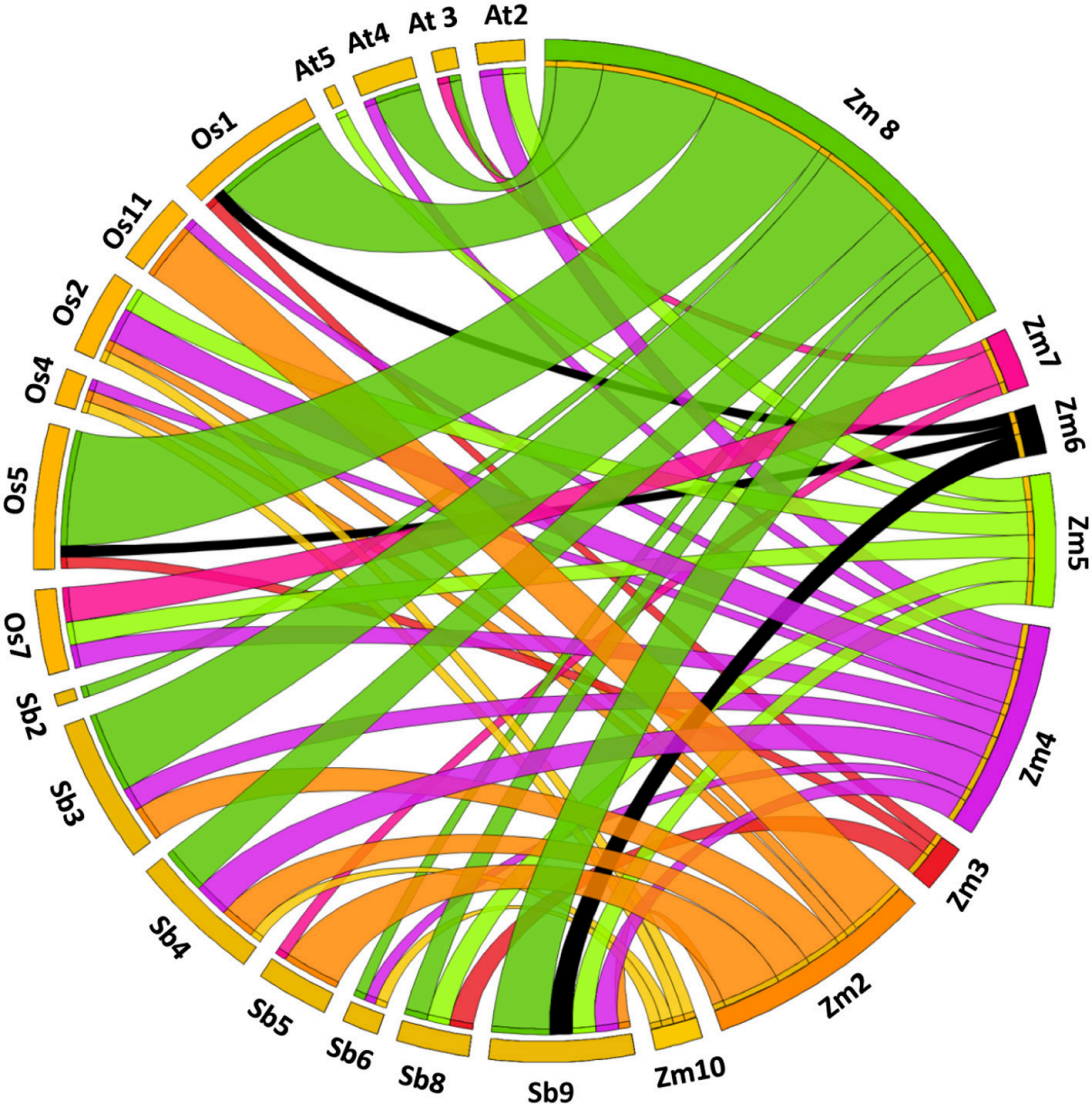
Syntenic relationship among CDPK genes mapped on different chromosomes belonging to *Arabidopsis*, maize, rice, and sorghum.

Divergence is an important process in the evolution of novel genes (Hughes, 1994). The synteny analysis helps in understanding of evolutionary and functional relationship between the orthologs. Our study revealed synteny of 28 genes with rice (87.5%), 26 genes with sorghum (81.25%) and 12 genes with *Arabidopsis* (40.62%). Maize showed maximum synteny with rice and lowest with *Arabidopsis* owing to the divergence during evolutionary time scale (Figure 5).

For paralog and ortholog gene pairs, we also estimated the time of divergence by examining ratio of synonymous (Ks) and non-synonymous (Ka) substitution. In total, we found 19 segmentally duplicated gene pairs while 30 tandems duplicated gene pairs in maize CDPK genes. More duplication events may be the reason for family expansion in maize (Cannon et al., 2004). In segmental duplication, Ka/Ks varied from 0.0 to 1.90 with an average of 0.28 while in tandem duplication, Ka/Ks ranged from 0.0 to 1.40 with an average of 0.32 (Supplementary Tables S2A,B). Similarly, in case of orthologous gene pairs, average Ka/Ks ratio for maize-*Arabidopsis*, maize-rice and maize-sorghum were 0.04, 0.07, and 0.08, respectively. Since Ka/Ks ratio for all the ortholog pairs was found to be <1, it is suggested that CDPK genes were evolved under purifying or stabilizing selection (Hurst, 2002; Yang et al., 2006) (Supplementary Table S2C–E).

### Amino acid motif analysis

A motif is a consensus or conserved region in protein or nucleotide sequences (Table 2). Among the five best identified motifs, motif 1 was the most frequently occurring one which was conserved in 234 proteins out of total 273 proteins, followed by motif 2 in 217 proteins. Motif 3 and 4 occurred equally in 211 proteins while the motif 5 was present in least number of proteins i.e., 156 proteins. Among different amino acids conserved in these five motifs, leucine (L) frequency was the highest (0.099), followed by Serine (0.089), Glycine (0.072), Alanine (0.068) etc. while minimum frequency was of tryptophan (W) amino acid (0.014). All the five identified motifs were part of the protein kinase domain.

**Table 2:** The best five protein conserved motifs identified using MEME software.

**Motif**	**Width**	**Best possible match**
1	37	HYSERHAAYFCQQIISVVEYCHSMGVCHRDLKPENFL
2	28	YGPEIDIWSCGCILYIMLCGVPPFWGET
3	39	DHYELGRKIGRGQFGVVYLCTCKETGETVACKKINKRKL
4	28	AKDLVRRMLVYDPKKRITAHEALCHPWF
5	28	CDFGLSKFFKPGEKFHDYVGSPYYVAPE

Motif 1, the most conserved domain in 234 proteins, represented protein kinase domain (ProteinkinaseDOM) and protein kinase C phosphorylation site domain (PKCPHOSPHOSITE) with a consensus pattern of [ST]-x-[RK], where S or T represented phosphorylation site. This site or domain preferred phosphorylation of serine or threonine residues which are available close to a C-terminal basic residue (Kishimoto et al., 1985; Woodgett et al., 1986). Motif 2 and motif 5 represented only protein kinase domain (ProteinkinaseDOM) that belongs to a very wide family of proteins which share a conserved catalytic core common to both serine/threonine and tyrosine protein kinases (Hanks and Hunter, 1995). The protein kinase domain have been found to play an important role in linking abiotic stress tolerance and metabolic responses of plants (Qin et al., 2011; Tao et al., 2014). The protein kinase domain is adjacent to the autoinhibitory or junction domain and consists of catalytic domain for ATP binding. This autoinhibitory domain alters during calcium binding which changes the protein conformation (Snedden and Fromm, 2001). The autoinhibitor domain acts as a pseudo substrate site and is found to be involved in activation of kinase (Harper et al., 1994). Interaction of autoinhibitor domain with calcium-binding domain regulates CDPKs, CaMKs, and SnRKs (Yoo and Harmon, 1996; Guo et al., 2001; Hook and Means, 2001; Huang and Huber, 2001; Soderling and Stull, 2001). In case of motif 3, Protein kinase ATP-binding region domain (ProteinkinaseATP) was found. This domain was functionally related with ATP binding and rich in glycine residues with lysine residue in vicinity, and located in the N-terminal of catalytic domain (Knighton et al., 1991). Motif 4 consists of one more extra site i.e., cAMP and cGMP-dependent protein kinase phosphorylation site (CAMPPHOSPHO-SITE) with a conserved pattern of [RK] (2)-x-[ST] where S or T represents phosphorylation site. cAMP and cGMP-dependent protein kinase are the key regulators of auxin transport and signaling (Christensen et al., 2000; Benjamins et al., 2001). This site shared preference for the phosphorylation of serine or threonine residues found close to at least two consecutive N-terminal basic residues (Glass et al., 1986). The statistical significance of motif prediction is correlated with biological significance and was an important reason for the motif analysis (Hart et al., 2000).

The N-terminal domains often contain palmitoylation and myristoylation sites for membrane attachment (Cheng et al., 2002). In our study, we found that out of 273 genes, only 59 genes contain myristoylation sites while all genes contain palmitoylation sites. The N-terminal acylation site was obtained in 181 sites (Supplementary Table S3). These sites play an important role in subcellular localization and function (Simeunovic et al., 2016). A total of 69 proteins have four EF-hand domains (Arabidopsis-16; maize-25; rice-13; sorghum-15) while 16 genes were having EF-hand domains <4 (Arabidopsis-4; maize-5; rice-6; sorghum-1). Less than 4 EF hand domains have previously been observed in monocot as well as dicot species. For example, in Arabidopsis, tomato, soybean, rice, grape, and maize 9, 4, 4, 1, 2, 4 members had <4 EF-hands, respectively (Cheng et al., 2002; Ray et al., 2007; Kong et al., 2013; Hettenhausen et al., 2016; Hu et al., 2016; Zhang et al., 2016).

### Gene expression analysis of CDPK gene family

CDPKs were found to be implicated in diverse physiological adaptations (Klimecka and Muszynska, 2007; Zhu et al., 2007; Xu et al., 2010). The expression profiles of genes indicate their functional relevance. A total of 27, 14, 23, and 16 genes were filtered in *Arabidopsis*, maize, rice and sorghum, respectively expressed in leaf, root, and shoot tissues. Previous researches have reported about the tissue-specific expression of CDPK genes (Frattini et al., 1999; Anil et al., 2000; Asano et al., 2002). Expression analysis showed highest expression in leaf tissue followed by root and stem. Similarly, higher expression of CDPK members was observed in growing tips of leaves, and coleoptiles of maize (Barker et al., 1998). In *Arabidopsis*, highest number of drought related genes were expressed in root (23) tissue followed by leaf (6) and shoot (5). In leaf (*Arabidopsis*), all CDPK genes were highly up regulated, while in root 11 genes were up-regulated and 12 genes down-regulated whereas only five genes were up-regulated in shoot. We noticed three common drought-related genes (*AtCDPK3, AtCDPK46*, and *AtCDPK54*) expressed in root and shoot of *Arabidopsis* but it was examined that these genes showed approximately twice the level of expression in leaf tissue as compared to root. Maize showed only up-regulation of genes in leaf (10), root (9) as well as shoot (3). No down-regulated expression of CDPK genes was found in maize. *ZmCDPK1* was expressed in leaf, root and shoot tissue but highest expression was noticed in leaf tissue followed by root and shoot. *ZmCDPK2, ZmCDPK3*, and *ZmCDPK4* showed almost similar expression in leaf and root. While, *ZmCDPK6* and *ZmCDPK9* showed higher drought-related expression in case of leaf as compared to shoot. In case of rice and sorghum, no significant drought expression was noticed in stem tissue. About 14 up-regulated and nine down-regulated genes were observed in leaf tissue of rice, while nine up-regulated and six down-regulated genes were expressed in leaf of sorghum. Similarly, in root tissue, only one up-regulated and one down-regulated gene were observed in rice as well as sorghum. These results suggested that the CDPK genes could play a differential role in various tissues and environments. In the leaf and root, *OsCDPK15* and *SbCDPK80* were down-regulated in rice and sorghum, respectively. Under drought stress, the role of calcium dependent kinases have been characterized in *Arabidopsis* as well as rice (Saijo et al., 2000; Zou et al., 2010).

**Figure 6:**
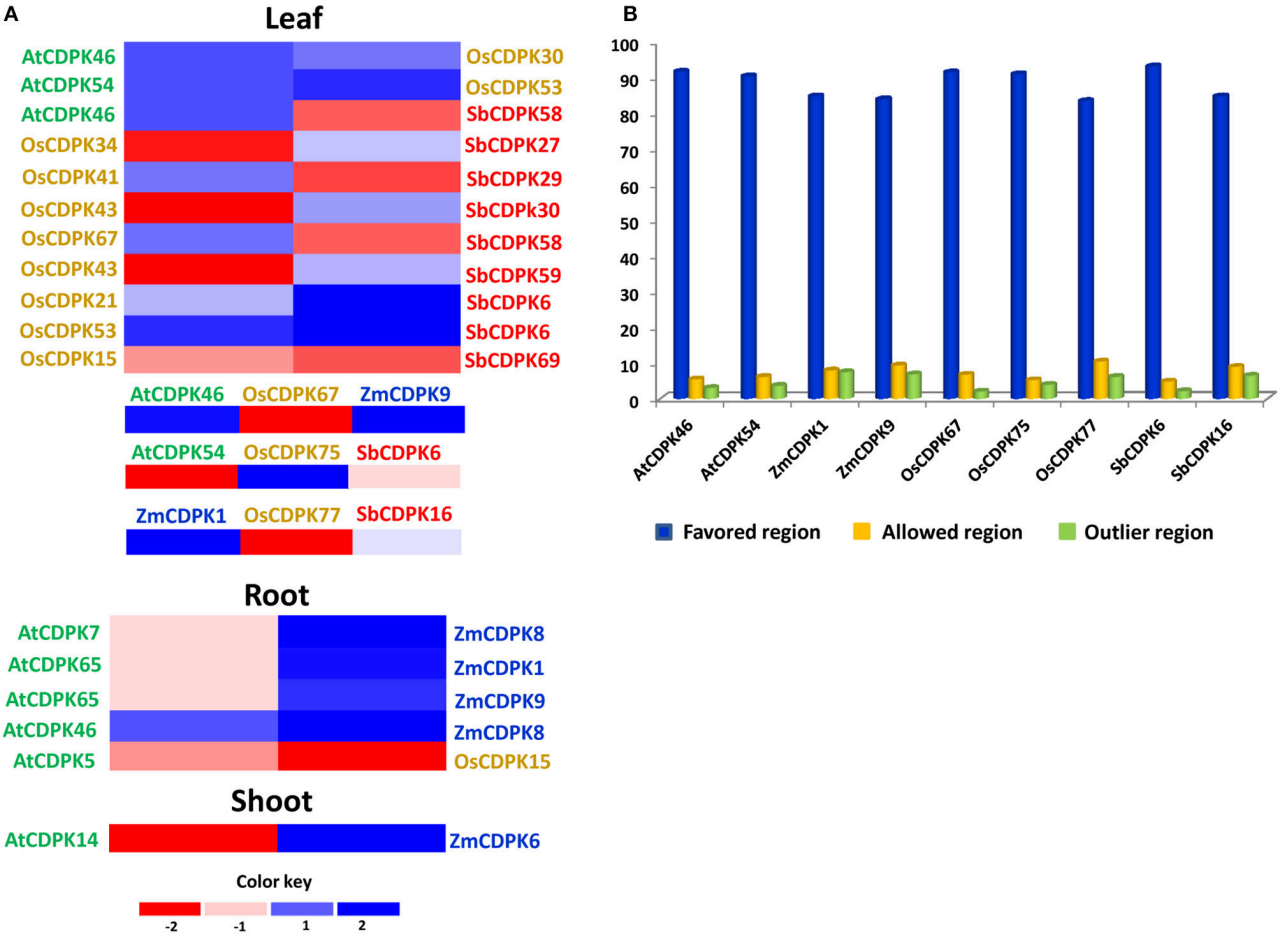
**(A)** Expression of drought-related ortholog pairs in different tissues (root, shoot, and leaf) where green, blue, golden, and red text represents *Arabidopsis*, maize, rice, and sorghum, respectively. **(B)** Ramachandran plot analysis of drought-related CDPK proteins using RAMPAGE describing percentage of residues in favored, allowed, and outlier region.

For comparative expression of CDPK genes, their orthologs were identified and compared in different tissues. *AtCDPK46*, up-regulated in leaf as well as root tissue was found to be orthologous to maize, rice and sorghum. *AtCDPK46*-*OsCDPK30, AtCDPK46*-*OsCDPK67* showed almost similar expression in leaf tissue while *AtCDPK46*-*SbCDPK58* showed contrasting expression. In *AtCDPK46*-*ZmCDPK8, AtCDPK46* retained its high expression in leaf while *ZmCDPK8* showed over-expression in root tissue indicating that *AtCDPK46* and its orthologs in other species are not species-specific or tissue-specific. Similarly, *AtCDPK54*, up-regulated in leaf under drought stress showed homology with rice and sorghum species. Ortholog pairs *AtCDPK54*-*OsCDPK75, AtCDPK54*-*OsCDPK53*, and *AtCDPK54*-*SbCDPK6* showed up- regulation in leaf tissue which represents tissue specific nature of these CDPK genes. Similar type of expression pattern in orthologous pairs suggests that they might have some conserved function during evolution. We also noticed some contrasting ortholog pairs. *AtCDPK14*, down-regulated in the shoot tissue of *Arabidopsis* while its ortholog *ZmCDPK6* was up-regulated in the shoot tissue of maize (Figure 6A). Similar results were also reported while studying orthology among CPK genes in wheat and rice. It was observed that most of wheat-rice ortholog pairs retained same type of expression except *TaCPK8*-*OsCPK1* and *TaCPK4*-*OsCPK24*, indicating species-specific functional evolution of ortholog pairs (Li et al., 2008).

*ZmCDPK1*, up-regulated in maize leaf tissue while its orthologs *OsCDPK77* (in rice) and *SbCDPK16* (in sorghum) were down and up-regulated respectively. *ZmCDPK9*-*OsCDPK67* pair was up-regulated in leaf tissue. While comparing rice and sorghum expression in leaf tissue, 10 ortholog pairs were identified in leaf tissue. In pair *OsCDPK15*-*SbCDPK69*, both CDPK genes were down- regulated while *OsCDPK21-SbCDPK6, OsCDPK53-SbCDPK6*, and *OsCDPK75-SbCDPK6* were up-regulated in leaf tissue in both species. In *OsCDPK34*-*SbCDPK27, OsCDPK41*-*SbCDPK29, OsCDPK43*-*SbCDPK30, OsCDPK43*-*SbCDPK59, OsCDPK67*-*SbCDPK58*, and *OsCDPK77*-*SbCDPK16*, expression was contrasting to each other. Such inconsistencies in expression levels between Arabidopsis, maize, rice, and sorghum CDPKs suggested that the regulatory control of particular kinases are likely species-specific.

Three contrasting drought-related orthologous groups (*AtCDPK46, OsCDPK67, SbCDPK6*), (*AtCDPK54, OsCDPK75, SbCDPK6)*, and (*ZmCDPK1, OsCDPK77, SbCDPK16*) expression were identified to be contrasting in leaf tissue. In first group, *OsCDPK67* was down-regulated; *AtCDPK46* and *SbCDPK6* were up regulated showing contrasting results. Similarly, in second and third group, *AtCDPK54* and *OsCDPK77* were down-regulated. Several maize-rice ortholog pairs maintained almost same expression as already reported- *ZmCPK11-OsCPK10, ZmCPK28-OsCPK19, ZmCPK29-OsCPK16, ZmCPK33-OsCPK*8 (Kong et al., 2013). In (*AtCDPK46, OsCDPK67, SbCDPK6*) pair, *AtCDPK46* and *SbCDPK6* have been found to be highly up regulated as compared to OsCDPK67 under drought stress. It has been noticed that under drought stress, *AtCDPK46* phosphorylates ABA-responsive bZIP-type transcription factors (ARF/AREB) via ABA-signaling. Similarly in (*AtCDPK54, OsCDPK75, SbCDPK6), OsCDPK75* has been found to phosphorylates and activates the OREB1, and plays vital role in ABA- signaling pathway. It provides drought tolerance by retaining plant growth under drought stress (Saijo et al., 2000).

### Secondary and tertiary structure of CDPK proteins

**Figure 7:**
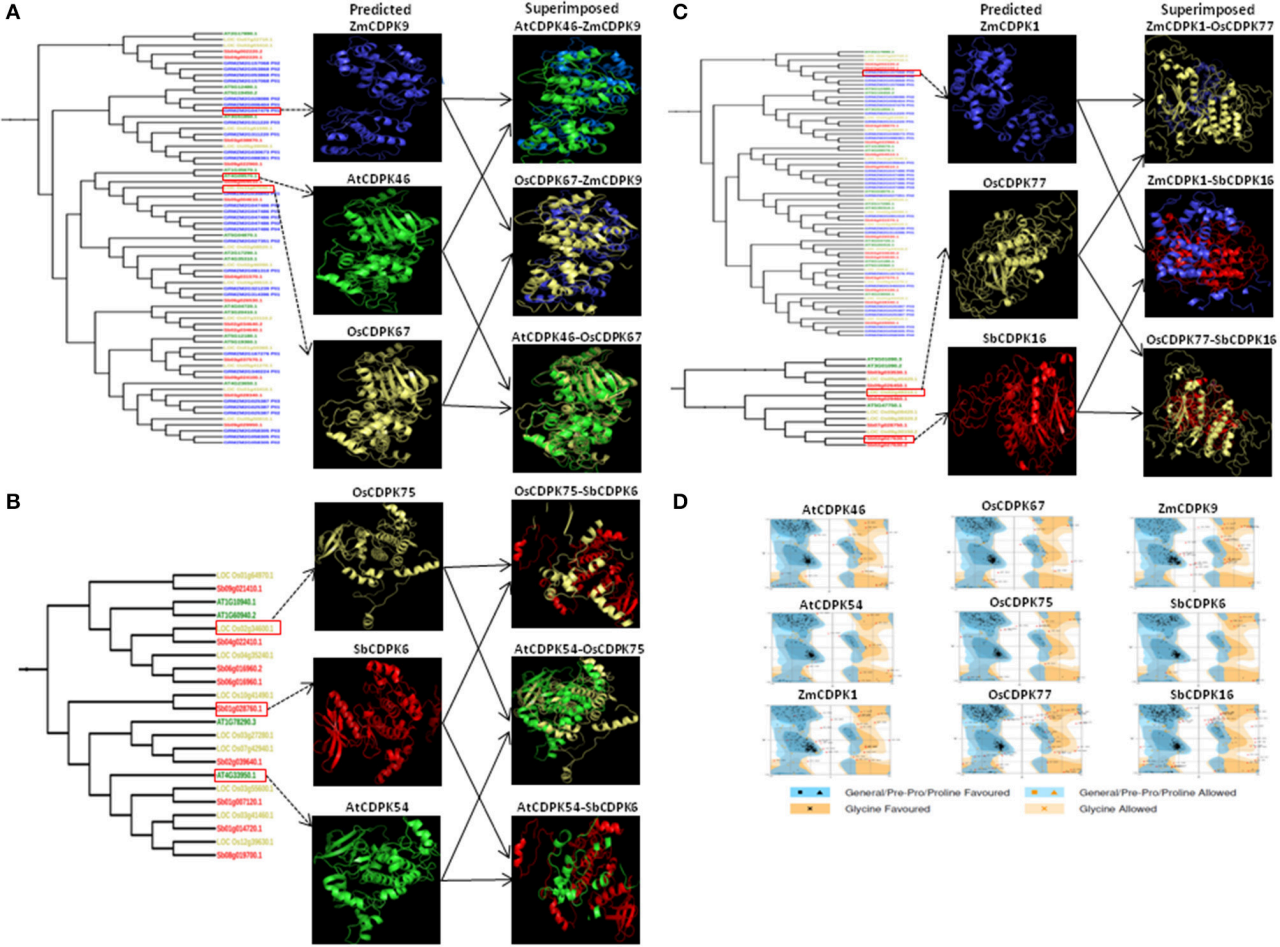
Predicted and superimposed 3-D structure of **(A)** *AtCDPK46, OsCDPK67*, and *ZmCDPK9* **(B)** *AtCDPK54, OsCDPK75*, and *SbCDPK6*, and **(C)** *ZmCDPK1, OsCDPK77*, and *SbCDPK16*. **(D)** Ramachandran plots of these nine selected structures using RAMPAGE.

For secondary and tertiary structure analysis of drought-related orthologous genes—*AtCDPK46, AtCDPK54, OsCDPK67, OsCDPK75, OsCDPK77, ZmCDPK1, ZmCDPK9, SbCDPK6*, and *SbCDPK16* were selected based on their differential expression in at least 3 species. Alpha helix, beta strand, transmembrane helix, and disordered regions were detected in these drought-related genes. Alpha helix was found to be most frequent followed by beta strand and transmembrane helix and disordered region (Table 3). The more frequency of the alpha helix revealed the higher level of conservation and stability of protein structure (Neelamathi et al., 2009). For proper understanding of protein functions and their interactions, 3-D structure is very important (Chen, 2009). The protein structure was generated by matching the target protein sequence to an evolutionary related known protein structure (template) (Kelley and Sternberg, 2009). Crystal structure of pbanka-031420 having PDB id 3Q5I was used as a template. According to our study, 91% (455) and 98% (354) residues of AtCDPK46 and AtCDPK54 were modeled at >90% accuracy. In case of ZmCDPK1and ZmCDPK9, 90% of the residues were modeled at >90% accuracy. In case of rice, 91, 55, and 99% of residues were modeled at >90% confidence level in OsCDPK67, OsCDPK77 and OsCDPK75, respectively, while in SbCDPK6 and SbCDPK16, 99% and 56% of residues, respectively were modeled (Figures 7A–C). In previous studies of *Hevea* spp. and *Arabidopsis* models, 83 and 86% residues of query sequence modeled with >90% confidence level with 29 and 26% disordered regions (Mathew et al., 2015).

**Table 3:** Secondary structure analysis of drought-related CDPK proteins using Phyre2 server.

**Species**	**Gene ID**	**Secondary structure**	**%**
*Arabidopsis*	AtCDPK46	Disordered	21.0
		Alpha helix	45.0
		Beta strand	14.0
		TM helix	3.0
	AtCDPK54	Disordered	19.0
		Alpha helix	39.0
		Beta strand	16.0
		TM helix	4.0
Maize	ZmCDPK1	Disordered	18.0
		Alpha helix	57.0
		Beta strand	4.0
		TM helix	5.0
	ZmCDPK9	Disordered	18.0
		Alpha helix	56.0
		Beta strand	5.0
		TM helix	5.0
Rice	OsCDPK67	Disordered	23.0
		Alpha helix	46.0
		Beta strand	13.0
		TM helix	3.0
	OsCDPK75	Disordered	23.0
		Alpha helix	44.0
		Beta strand	14.0
		TM helix	4.0
	OsCDPK77	Disordered	49.0
		Alpha helix	26.0
		Beta strand	9.0
		TM helix	0.0
Sorghum	SbCDPK6	Disordered	17.0
		Alpha helix	42.0
		Beta strand	17.0
		TM helix	5.0
	SbCDPK16	Disordered	48.0
		Alpha helix	23.0
		Beta strand	8.0
		TM helix	0.0

### 3-D protein structure evaluation

The predicted 3-D structures of AtCDPK46, AtCDPK54, ZmCDPK1, ZmCDPK9, OsCDPK67, OsCDPK75, OsCDPK77, SbCDPK6, and SbCDPK16 were evaluated for their quality by Ramachandran plot analysis, ANOLEA, ProSA program, and Verify—3D score. To know about the stereo chemical quality of side chains and polypeptide backbone, Ramachandran plot analysis was examined using RAMPAGE. To examine the reliability of the target proteins, percentage quality measurement and torsion angles Ø and Ψ was used, in which three regions were known “favored,” “allowed,” and “outlier regions.” These values were calculated for these nine-selected droughts responsive proteins (Figure 7D). Altogether we determined that in AtCDPK46 (97%), AtCDPK54 (96.4%), ZmCDPK1 (92.6%), ZmCDPK9 (93.2%), OsCDPK67 (98.1%), OsCDPK75 (96.1), OsCDPK77 (93.8%), SbCDPK6 (97.9%), and SbCDPK16 (92.6%), approximately most of the residues lie in favored and allowed region of Ramachandran plot suggesting that all the predicted 3-D structure of CDPK proteins are good quality structures (Figure 6B).

Verify-3D checks the protein by analyzing compatibility of protein model (3-D) with its amino acid sequence (1D). Score between 0.1 and 0.71 is considered as a good score of the residues and the profile score above zero indicates acceptance of side chain environment (Eisenberg et al., 1997). Verify-3D results showed 79.64, 78.45, 56.90, 58.03, 91.23, 72.33, 60.88, 68.77, and 61.01% of AtCDPK46, AtCDPK54, ZmCDPK1, ZmCDPK9, OsCDPK67, OsCDPK75, OsCDPK77, SbCDPK6, and SbCDPK16, respectively had an averaged profile score ≥ 0.2. ANOLEA program calculates energies of the protein chains in the form of high energy (%) and Z-score (Melo and Feytmans, 1998). Z-score is inversely related with the reliability i.e., lower Z-score indicates high reliability and vice-versa. Z-scores calculated by ANOLEA were 3.68, 5.66, 18.89, 15.98, 3.84, 5.17, 5.90, 3.37, and 6.20 for AtCDPK46, AtCDPK54, ZmCDPK1, ZmCDPK9, OsCDPK67, OsCDPK75, OsCDPK77, SbCDPK6, and SbCDPK16, respectively. This program estimates errors, global quality of the predicted protein structure and also energy values for each amino acid (Melo and Feytmans, 1998; Offredi et al., 2004). The 3D-1D score, ProSA and ANOLEA scores validated our structures to be a good quality model.

ProSA server on the basis of distance based pair potential calculates interaction energy per residue. Score of overall quality model of AtCDPK46, AtCDPK54, ZmCDPK1, ZmCDPK9, OsCDPK67, OsCDPK75, OsCDPK77, SbCDPK6, and SbCDPK16 were −10.43, −6.28, −6.71, −6.87, −9.3, −5.22, −5.04, −5.73, and −6.24, respectively. The reliability of these predicted models of drought-related proteins can be confirmed by negative ProSA energies of the residues. ProSA analysis confirmed that the folding energy of the predicted 3-D structure was in agreement with the template model (Table 4).

**Table 4:** Evaluation of 3-D protein structure using ANOLEA, Verify-3D, and ProSA.

**Protein**	**Evaluation program**	**Overall model quality**	**Z-score**	**3D-1D profile**
AtCDPK45	ANOLEA	–	3.68	–
	Verify-3D	–	–	79.64 ≥ 0.2
	ProSA	−10.43	–	–
AtCDPK54	ANOLEA	–	5.66	–
	Verify-3D	–	–	78.45 ≥ 0.2
	ProSA	−6.28	–	–
ZmCDPK1	ANOLEA	–	18.89	–
	Verify-3D	–	–	56.90 ≥ 0.2
	ProSA	−6.71	–	–
ZmCDPK9	ANOLEA	–	15.98	–
	Verify-3D	–	–	58.03 ≥ 0.2
	ProSA	−6.87	–	–
OsCDPK67	ANOLEA	–	3.84	–
	Verify-3D	–	–	91.23 ≥ 0.2
	ProSA	−9.3	–	–
OsCDPK75	ANOLEA	–	5.17	–
	Verify-3D	–	–	72.33 ≥ 0.2
	ProSA	−5.22	–	–
OsCDPK77	ANOLEA	–	5.9	–
	Verify-3D	–	–	60.88 ≥ 0.2
	ProSA	−5.04	–	–
SbCDPK6	ANOLEA	–	3.37	–
	Verify-3D	–	–	68.77 ≥ 0.2
	ProSA	−5.73	–	–
SbCDPK16	ANOLEA	–	6.2	–
	Verify-3D	–	–	61.01 ≥ 0.2
	ProSA	−6.24	–	–

### Structure alignment and superimposition

To have an idea about structural variation in drought-related CDPK proteins among different species i.e., *Arabidopsis*, maize, rice, sorghum, and 3-D structures were superimposed on each other (Figures 7A–C). The root mean square deviation (RMSD) and TM-score was calculated for each superimposed structure. RMSD is measured by the averaging distance between the backbones of superimposed structure (Pettersen et al., 2004). Previously reported that RMSD score between 1 and 2Å represents closely related proteins while RMSD score <6 indicates likely related proteins. While TM-score between 0.0 and 0.30 indicates random similarity between superimposed structures while if TM-score lies between 0.5 and 1.0, it suggests that the superimposed proteins may have same folds. The RMSD scores for all the ortholog pairs were <6 (AtCDPK46-OsCDPK67, AtCDPK46-ZmCDPK9, OsCDPK67-ZmCDPK9, AtCDPK54-OsCDPK75, AtCDPK54-SbCDPK6, OsCDPK75-SbCDPK6, ZmCDPK1-OsCDPK77, ZmCDPK1-SbCDPK16, and OsCDPK77-SbCDPK16) indicated these proteins were structurally similar to each other. Similarly, TM-score for all these 9 superimposed structures was between 0.5 and 1 indicating that the ortholog pairs had similar folding pattern. Some structural differences between the superimposed structures may be due to insertion or deletion in different loop regions. Those structures which possess low RMSD score, shows high structural similarity and common folding pattern with the template model while high RMSD score indicates low quality model. As already reported, *Hevea* CDPK model was significantly superimposed on *Arabidopsis* CDPK21 model suggesting orthologous nature of genes. Proteins with same architecture and domains possess common functional features (Forslund et al., 2011).

## Conclusion

The present study was the first attempt to focus the comparative structural and functional characterization of CDPK proteins in *Arabidopsis*, maize, rice and sorghum. CDPK genes revealed more intron-rich regions (Type-II) over intron-poor (Type-I) regions lead in to alternative splicing and exon shuffling. This phenomenon could be the major reason for the functional diversity of CDPKs responses to various stress signals. The most frequently occurred motif in all the species contained the protein kinase and protein kinase C phosphorylation site domains. Pfam domain structure similarity among genes of specific subgroups in phylogenetic tree suggested that the genes were structurally conserved in the subgroups. Phylogenetic analysis of CDPK proteins revealed that the Group III was the highly conserved group during the evolution. Our study identified that the duplication events could be the reason for the expansion of CDPK genes. Maize-rice species genes were less divergent as compared to maize-sorghum and maize-*Arabidopsis*. The maximum synteny was observed between maize-rice, and the minimum was between maize-*Arabidopsis* owing to the divergence during evolutionary time scale. Tissue-specific and across-tissues expression of drought-responsive genes in different species could be exploited in modulation of drought tolerance mechanism in crop plants. Predicted 3-D structures of a set of selected nine CDPK proteins suggested the both divergence and similarity among CDPK proteins. Superimposed 3-D structures of orthologous drought proteins revealed similar folding pattern indicating the structural and functional conservation across the species. Many of the droughts-responsive CDPKs were associated with regulation of physiological and molecular pathways to impart the drought tolerance. The information from the present investigation could be useful in understanding of the tolerance mechanism and development of drought tolerant genotypes in maize as well as in rice and sorghum. Our results from the comparative analysis of CDPK genes will further be extended to the stress breeding programmes of other related species.

## Author contributions

NT and SM conceived and designed the experiments; SM, MGM, ARR, PAJ, and PD analysed the data. NT, SM, MGM, and PD drafted the manuscript. All authors read and approved the final manuscript.

### Conflict of interest statement

The authors declare that the research was conducted in the absence of any commercial or financial relationships that could be construed as a potential conflict of interest. The reviewers, MK, SK, and handling Editor declared their shared affiliation.
