# Supplementary figures and images for: Comparative Analysis of CDPK Family in Maize, Arabidopsis, Rice, and Sorghum Revealed Potential Targets for Drought Tolerance Improvement
Source: Front Chem. 2017 Dec 19;5:115. doi: 10.3389/fchem.2017.00115 (PMC5742180; doi:10.3389/fchem.2017.00115)

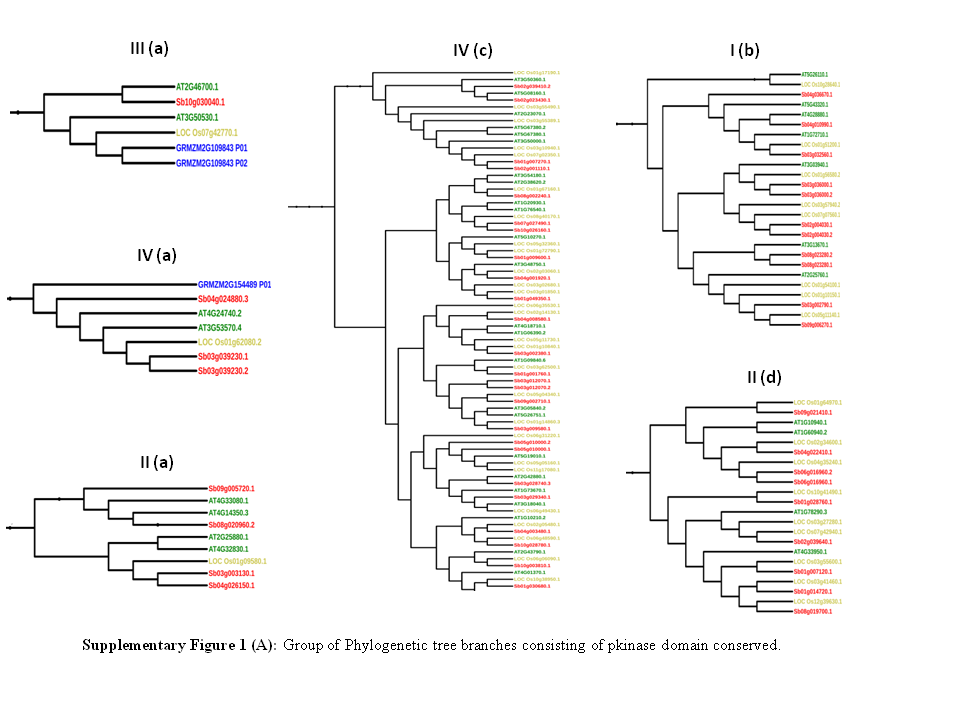

Supplement: Supplementary file 4 [file Image1.TIF]

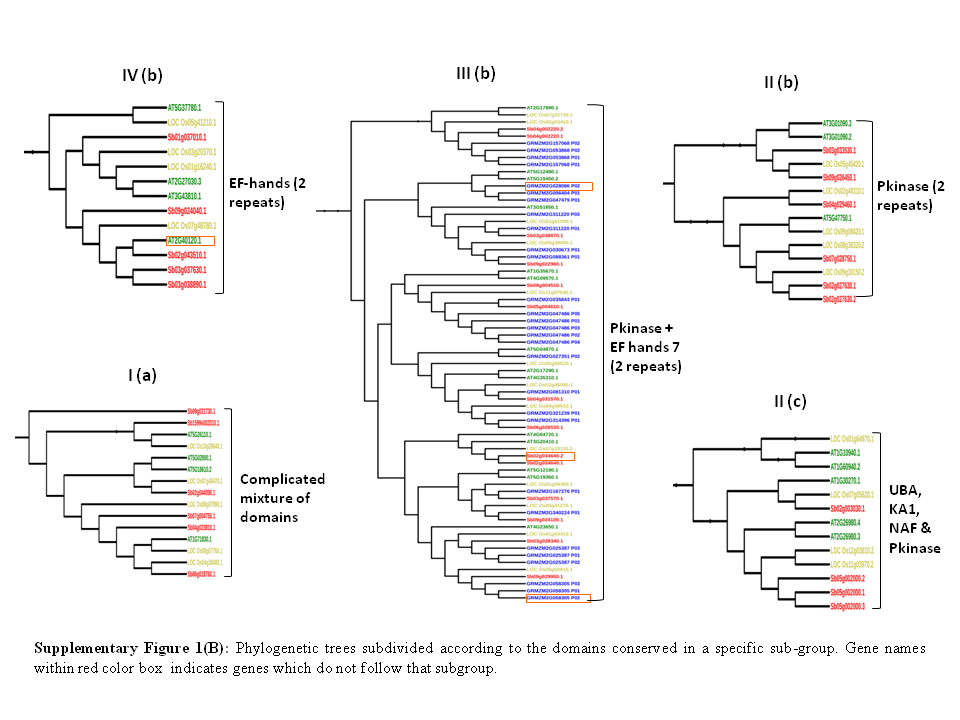

Supplement: Supplementary file 5 [file Image2.TIF]
